# Exploring the involvement of the alternative complement pathway in non-infectious uveitis pathogenesis

**DOI:** 10.3389/fimmu.2023.1222998

**Published:** 2023-12-08

**Authors:** Prerna Kulshrestha, Pallavi Goel, Somasheila Murthy, Mudit Tyagi, Soumvaya Basu, Pratik Gogri, Inderjeet Kaur

**Affiliations:** ^1^ Prof. Brien Holden Eye Research Centre, L. V. Prasad Eye Institute, Hyderabad, India; ^2^ School of Life Science, Manipal Academy of Higher Education, Manipal, India; ^3^ Shantilal Shanghvi Cornea Institute, L. V. Prasad Eye Institute, Hyderabad, India; ^4^ Smt. Kannuri Santhamma Centre for Vitreo Retinal Diseases, L. V. Prasad Eye Institute, Hyderabad, India

**Keywords:** uveitis, complement pathway, inflammation, miRNA, aqueous humor, vitreous humor

## Abstract

**Purpose:**

Non-infectious uveitis is a complex disease characterized by intraocular inflammation of the uveal area and the leading cause of vision impairment and blindness in young people globally. However, what triggers inflammation and contributes to its recurrence remains unclear. The complement system has been linked to various immunological and inflammatory conditions. In the present study, we have systematically evaluated the role of the alternative complement pathway in the pathogenesis of non-infectious uveitis.

**Methodology:**

Quantitative PCR was done in the peripheral leukocytes to study the expression of genes and regulatory miRNA in both anterior and posterior uveitis (n=28 in each category). Multiplex ELISA was performed to measure alternative pathway complement components, such as C3b, factor B, and CFH, and aqueous humor of infectious and non-infectious uveitis patients and non-inflammatory controls (n=10 each). Western blotting was done to validate the ELISA findings in a subset of patients and controls.

**Results:**

Downregulation of *C3* and *CFH* mRNA in the peripheral blood was shown by quantitative PCR in the group of anterior uveiits (AU), while the opposite result was found in the group of posterior uveitis (PU). ELISA levels of C3b and CFH proteins were significantly higher in aqueous humor of infectious and non-infectious uveitis (*p = 0.03 and **p = 0.0007 respectively) as compared to the control group. Western blotting further validated (VitH) the activation of the complement cascade in the aqueous (AH) and vitreous humor of patients with non-infectious uveitis, with an increased level of C3b (n=6) and CFH (n=4) in aqueous humor. C3b level was significantly increased while CFH was reduced relative to controls in the vitreous humor (VitH) of posterior uveitis patients compared to controls (n=27 in each category). A C3b to CFH ratio was computed to assess the regulation of complement activation and this index was several folds higher in both anterior and posterior uveitis (n=10 each). The expression of miRNA-hsa-miR-146a and miRNA-hsa-miR-155-5p that regulates CFH was downregulated and nicely correlated with the increased complement proteins in both anterior and posterior uveitis (n=10 each).

**Conclusion:**

Our results demonstrate a clear role of CFH and the activation of the alternative complement pathway in the pathogenesis of non-infectious uveitis; however, its therapeutic potential warrants further investigations.

## Introduction

Uveitis is a major sight-threatening disorder. The term uveitis defines the process of inflammation in the uvea area of the eye, which includes the iris, ciliary body, and choroid. However, if uveitis remains untreated it can affect any area of the eye. Anatomically, uveitis can be divided into four categories primarily based on the location of inflammation in the eye, i.e., anterior, intermediate, posterior, and pan-uveitis (1). Symptoms and consequences can range from pain, photophobia, watering, and redness to complete vision loss. It can be further divided into two subtypes: infectious (IU) and non-infectious uveitis (NIU). Bacteria, viruses, or any other infectious agent may induce infectious uveitis (2). While non-infectious uveitis can be largely autoimmune or idiopathic in nature, it is also reported to be associated with other systemic conditions (e.g., sarcoidosis and Behcet’s disease) that are known to result from immune dysregulation (3, 4).

Non-infectious uveitis is usually T cell–mediated and includes a heterogeneous group of sight-threatening immune-mediated ocular and systemic disorders. (e.g., sarcoidosis and Behcet’s disease). While the pathophysiology of non-infectious uveitis is not completely clear, it is assumed that it could be a multifactorial autoimmune disease, or caused by an imbalance of anti-inflammatory and inflammatory cells that leads to the release of several cytokines. These released cytokines further recruit leucocytes and neutrophils from the circulation and enhance inflammation, causing tissue damage (5–7). While the exact trigger and cause of the disease remain unknown, many studies based on different animal models showed that autoimmune responses to various ocular antigens triggered inflammation, as seen in non-infectious uveitis (8–11). Some recent studies that were focused on the underlying mechanisms of uveitis, indicated that complement proteins could be involved in the pathogenesis of uveitis. Besides, the IgG-containing circulating immune complexes in patients with uveitis were found to have significantly increased levels of C3d (12). A study showed that depletion of the host’s complement system resulted in the complete inhibition of experimental autoimmune anterior uveitis (EAAU) (13). Introducing the soluble complement inhibitor (sCrry) was also shown to suppress EAU (14). Elevated levels of C3a, C3c, and CFB proteins have also been reported in the aqueous humor of uveitis patients (15, 16). While these studies have indicated the role of complement proteins in uveitis associated with other related pathologies, their exact role in non-infectious uveitis remains unclear. Complement factor H (CFH) is a potent inhibitor of the C3 alternative complement pathway, and its role has also not been explored in detail. Furthermore, it would be worthwhile to study the epigenetic regulation of complement pathway genes by miRNA that act by binding to the complementary regions of the target gene, causing either its suppression or degradation. MiR-155 and miR-146 are known to regulate inflammation and autoimmunity. Increased miR-146a could enhance the expression of *IL-1β* and suppress the expression of *CFH* in the hippocampi of chronic temporal lobe epilepsy rat models, whereas reduced miR-146a could suppress the expression of *IL-1β* and enhance the expression of *CFH* (17). In the context of autoimmune diseases, such as Bechet diseases, the downregulation of both these microRNAs was seen in the blood samples of patients as compared to controls. While miR-146a was significantly downregulated, miR-155-5p showed a downregulation that was not statistically significant (18). Another study, conducted by Zhou et al., also reported a reduced expression of miR-146a and miR-155-5p that further contributed to an abnormal Treg phenotype in patients with active rheumatoid arthritis (RA). It was also observed that the pro-inflammatory effect of reduced miR-146a expression prevails over the counteracting impact of reduced miR-155-5p expression, leading to pro-inflammatory alterations of the phenotype of Tregs in RA (19).

Based on all this evidence, we proposed if the alternative complement pathway has a major role in the pathogenesis of human non-infectious uveitis. We also evaluated the estimation of major alternative complement pathway components, namely, C3 and CFH, in the aqueous and vitreous humor of anterior and posterior uveitis patients, as well as their expression and regulation by miRNAs. The results from this study are expected to expand and better our understanding of the disease pathophysiology and provide possible therapeutic manipulation of complement activity for checking inflammation and disease progression/recurrence.

## Materials and methods

### Enrollment of study participants and sample preparation

The study adhered to the tenets of the Declaration of Helsinki and was approved by the Institutional Review Board of L V Prasad Eye Institute, Hyderabad, India (Ethical reference no. LEC-09-18-134). The study cohort comprised a total of 188 samples. The inclusion criteria for the patients with uveitis were diagnosis based on a standardized set of criteria as per the SUN classification system for grading the four aspects of intraocular inflammation (“Standardization of Uveitis Nomenclature for Reporting Clinical Data. Results of the First International Workshop,” 2005). Active uveitis is defined as having > 1+ AC cells and/or a vitreous haze score (SUN scale) and other signs of activity such as choroiditis, macular edema, and vasculitis. Anterior uveitis is defined based on the anterior chamber as the site(s) of inflammation, while posterior uveitis is defined based on the retina or choroid as the primary site of inflammation. Infectious and non-infectious uveitis were distinguished based on specific ocular signs, review of systems (by internists), and specific laboratory investigations (wherever applicable). These were broadly based on the Standardization of Uveitis Nomenclature for Disease Classification Criteria (SUN Part 2) for different uveitis entities. The aqueous humor samples were taken from patients who had cataract surgery, namely, from the control group. The control vitreous humor samples were obtained from individuals who were having macular hole surgery or surgery for rhegmatogenous retinal detachment.

Medical therapy for non-infectious and infectious uveitis: All patients with anterior uveitis received topical prednisolone acetate 1% eyedrops, instilled every 2 to 4 hours in the first week after diagnosis and then tapered over the next 6 to 8 weeks. Atropine (1%) or homatropine (2%) eye drops were added as cycloplegic agents. All patients were evaluated by a physician and were cleared medically to receive oral immunosuppression. The topical medication was discontinued if cells were absent at the end of 6-8 weeks. In the case of intermediate and posterior uveitis and recalcitrant anterior uveitis, oral corticosteroid medication (prednisolone) was administered in the dose of 1mg/kg body weight at the time of diagnosis and then tapered every week or based on clinical response.

Immunomodulator therapy was started with oral methotrexate at the dose of 10 mg once a week and was stepped up to a maximum dose of 20 mg once a week as necessitated for control of inflammation on a case-to-case basis. The indications for oral immunomodulators were: 1) as a steroid-sparing agent; 2) where the disease mandated more than 2 to 3 months of immunosuppressive therapy; 3) when the disease at onset was severe and the treating physician anticipated a prolonged course; 4) as immunosuppressants in patients intolerant to oral steroids; and 5) as additional inflammatory control in cases where the inflammation did not subside at the end of 3 months. The duration of the immunomodulator therapy ranged from 6 months to 2 years.

All cases of infectious uveitis (presumed ocular tuberculosis) were started on four drug-antitubercular therapy (isoniazid and rifampin for two months and ethambutol and pyrazinamide for 9 months, dosage as per body weight) as per the protocol for extra-pulmonary tuberculosis.

The side effects of all the medications were evaluated by periodically monitoring serological parameters such as complete blood counts, liver function tests, and renal function tests. 

### Biological sample collection

A prior informed written consent was taken from the patients undergoing cataract surgery. To remove any kind of cellular debris, aqueous humor samples were centrifuged at 12,000 rpm for 10 min at 4°C. The supernatant was collected, aliquoted, and stored at -80°C for further experiments. Similarly, vitreous humor samples (100 μl) were collected from patients with uveitis undergoing vitrectomy. There were 57 patients and 47 healthy controls. The demographical details of the subjects used for protein and gene expression are given in **Supplementary Tables 1**-**4**. All the samples were processed in the same manner as mentioned above to remove any cellular debris and the supernatant collected was aliquoted and stored at -80°C for further experiments. Protein lysis was performed by adding an equal volume of RIPA buffer followed by precipitation using ice-cold acetone overnight at -80°C. On the following day, the protein lysate was centrifuged at 13,000 rpm for an hour and protein pellets were dissolved in RIPA buffer. The total protein concentration was estimated by bicinchoninic acid (BCA) assay. For quantification of mRNA and miRNA expression, the blood samples were collected in K3EDTA-coated vacutainers from patients with anterior uveitis (n=28), patients with posterior uveitis (n=28), and controls (n=28). The blood samples were further processed for RNA using a kit method as described in the next section below.

### RNA isolation and quantitative real-time PCR for mRNA and miRNA

Total RNA was isolated by using a Pure link mini-RNA isolation kit (Catalog no. 12183018A; Thermo Fisher Scientific). The quantity of RNA was measured using nanodrop. In all, 600ng of total RNA was converted into cDNA using the verso cDNA conversion kit (Catalog no. AB1453B; Thermo Fisher Scientific) as per the manufacturer’s protocol. Quantitative real-time PCR was performed on a 7900 HT platform using SYBR green (F-416 Thermo Fisher Scientific) chemistry for *C3*, *CFH, IL6, IL4, IL1β*, and *CD11b*. *β-actin* was used as the internal control. For miRNA, 600ng of total RNA was converted into cDNA using the miScript II RT Kit (Catalog no. 218161; Qiagen) as per the manufacturer’s instructions. quantitative PCR was performed on the same platform again using the SYBR green chemistry by miScript SYBR Green PCR Kit (Catalog no. 218173; Qiagen) for hsa-miR146a-5p and hsa-miR155-5p. U6 was used as the internal control. Primer sequences for mRNA and miRNA are provided in **Supplementary Tables 5** and **6**. The cycle threshold (Ct) values calculated for each test gene and miRNA were obtained for each sample using SDS2.3 software, and fold change was calculated using 2^−ΔΔCt^. A p-value of less than 0.05 was considered significant.

### MiRNA-mRNA target interaction network

The interaction network for MiRNA, alternative complement components, and inflammatory gene targets was made with the help of miRNet 2.0 software (https://www.mirnet.ca/).

### Enzyme-linked immunosorbent assay

A multiplex ELISA assay (Catalog no. HCMP2MAG-19K-03; MERCK Millipore) was used to assess the differential expression of C3b, CFH, and CFB (complement factor B) in aqueous humor samples of patients with non-infectious anterior uveitis (n=10), patients with infectious anterior uveitis (n=10), and controls (n=10) following the manufacturer’s protocol. To begin with, each sample (20 µl) was diluted with 40 µl of assay buffer in a ratio of 1:3. The standards were prepared by adding 250 µL of deionized water. The 96-well plate containing the suspension captured the antibody coated onto the bead. Samples were incubated with beads specific for cytokines of interest in a single well of a 96-well plate followed by washing steps to remove unbounded antibodies. Biotinylated detection antibodies were added to each well containing samples and then Streptavidin-Phycoerythrin (Strep-PE) antibody was added, binding to the biotinylated detection antibodies. The remaining wells were assigned for the standard controls containing a known amount of each cytokine. The plate was scanned on MAGPIX^®^ with xPONENT v4.3 software.

### Western blotting

The protein expression of C3 and CFH in the aqueous and vitreous humor samples of non-infectious uveitis and healthy patients was further validated by western blotting (Ms-C3, Catalog no. sc-28294, Santacruz; Ms-CFH, Catalog no. sc-166613, Santacruz). Under non-reducing conditions, the activated proteolytic fragments of C3 were observed in the aqueous and vitreous humor of the non**-**infectious uveitis and control subjects (Ms-C3, Catalog No. sc-28294, Santacruz**).** The details of the concentration of proteins and antibody dilutions are given in **Supplementary Table 7**.

SDS-PAGE was used to separate the protein samples. For the analysis, 15 µg of protein was loaded for C3 detection, and 30 µg of protein was loaded for CFH detection in separate wells/gel. Then, the separated proteins on the gel were transferred to a PVDF membrane (Catalog no. IPFL00010; Millipore) at a constant voltage of 25 V by wet transfer for a period of 1–2 hrs. Ponceau staining was done to determine equal loading of proteins. Furthermore, the primary antibody was added and incubated overnight at 4°C. After incubation was done, the blot was washed with PBST and 1x-PBS thrice and incubated with secondary antibodies (anti-Ms. 680RD, Catalog no. 926–68070, LI-COR, anti-Rb 800CW, Catalog no.926–32211, LI-COR) for 1 hr at room temperature. The blot was scanned, and bands were visualized using a LI-COR image scanner. The band intensities were quantified using Image J software (v1.4.3.x).

### Statistical analysis

The average band intensity for each protein in each group was calculated and a bar graph was plotted. A t-test was performed to estimate the significance between cases and controls. A p-value ≤ 0.05 was considered significant.

## Results

### Quantitative assessment of the alternative complement pathway in non-infectious anterior uveitis

**Figure 1:**
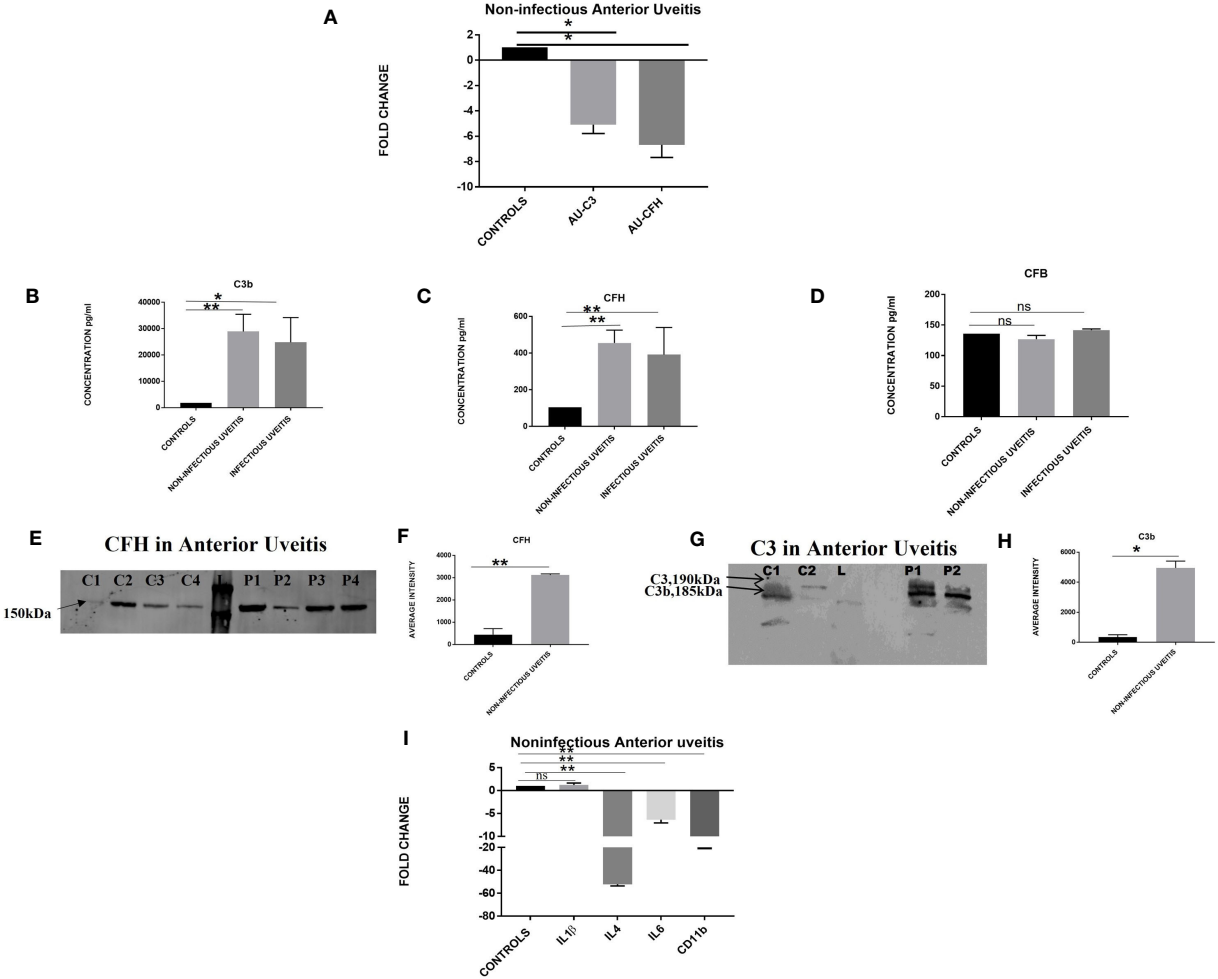
**(A)** Differential expression based on quantitative PCR for complement *C3* and *CFH* in non-infectious anterior uveitis category (n=18) vs. non-inflammatory controls (n=18), (AU corresponds to non-infectious anterior uveitis). Histogram showing the quantitative estimation of complement components **(B)** C3b, **(C)** CFH, **(D)** CFB in the aqueous humor samples based on multiplex ELISA from non-infectious uveitis (n=10), infectious uveitis (n=10) vs. non-inflammatory controls (n=10). **(E)** Representative western blot of total CFH in aqueous humor of non-infectious uveitis and non-inflammatory controls. **(F)** Graph representing the quantification of total CFH in aqueous humor of non-infectious uveitis and non-inflammatory controls by densitometry (NIU, n=4 and controls, n=4). **(G)** Representative western blot of total C3b in aqueous humor of non-infectious uveitis and non-inflammatory controls. **(H)** Graph representing the quantification of total C3b in aqueous humor of non-infectious uveitis and non-inflammatory controls by densitometry (NIU, n=6 and controls, n=6). **(I)** Differential gene expression based on quantitative PCR for non-infectious anterior uveitis category (n=15) vs. non-inflammatory controls (n=15). In the western blotting experiments, C represents control aqueous humor; P, NIU aqueous humor; L, protein ladder. For all the experiments data are represented as mean ± SEM. *p ≤ 0.05, **p ≤ 0.01, ns, p > 0.05.

Quantitative real-time PCR was performed for the candidate genes, i.e., *C3* and *CFH*, in the peripheral leukocytes of patients with non-infectious anterior uveitis and control subjects (**Figure 1**). The expression of *C3* and *CFH* genes was significantly downregulated in the patients with non-infectious uveitis as compared to controls (*C3*, AAU vs. controls, FC -5.0 ± 0.7; ^*^ *p* = 0.03; *CFH* AAU vs. controls, FC -6.67 ± 1.0; *p = 0.04). An enzyme-linked immunosorbent assay (ELISA), performed for the assessment of complement activation in the aqueous humor of patients with anterior uveitis, showed significantly higher expression of activated fragment of complement component C3b in non-infectious (28906.0 ± 6556; **p = 0.0007) and infectious uveitis (24823.2 ± 9382; *p = 0.03) as compared to controls (1868± 1439). In addition, a significant increase in the level of CFH was also observed in the non-infectious (455± 70; **p = 0.0001) and infectious uveitis (391 ± 147; *p =0.004) as compared to controls (104.3 ± 18.2). However, a non-significant difference was seen for the CFB levels among both non-infectious (126.8 ± 6.2; *p* = 0.3) and infectious uveitis (141.5 ± 53; *p* = 0.5) vs. controls (135 ± 6.2). The validation of the multiplex ELISA results by western blotting confirmed multiple bands of activated C3b (185 kDa) and a few others in the patients with non-infectious uveitis and in controls. However, the intensity of these bands was significantly higher in anterior NIU patients compared to controls (NIU, 4952.5 ± 452.5; *p = 0.01; Controls, 889 ± 348.1). A higher expression of CFH was also observed in the anterior NIU patients as compared to controls (NIU, 3119 ± 49.6; *p = 0.002); (Controls, 441.6 ± 1.5). Since complement activation is known to activate microglia leading to an increased expression of pro-inflammatory cytokines, expressions of cytokines genes and *CD11b*, a marker of activated microglia, were analyzed subsequently among patients with non-infectious anterior uveitis vs. controls. Quantitative real-time PCR was performed for several interleukin genes including *IL6, IL4, IL1β*, and *CD11b* in the peripheral leukocytes and showed an upregulation of pro-inflammatory cytokine *IL1β* (AAU vs. control, FC 1.22 ± 0.4; *p* = 0.6) and significant downregulation of *IL6* (AAU vs. control, FC-6.3 ± 0.7; **p = 0.006), *CD11b* (AAU vs. control, FC-18.8 ± 1.2; **p = 0.004), and anti-inflammatory *IL4* (AAU vs. control, FC-52 ± 1.4; ** *p* = 0.005) among NIU patients.

### Are there any specific regulators that mediate the aberrant alternative complement pathway activation in uveitis?

**Figure 2:**
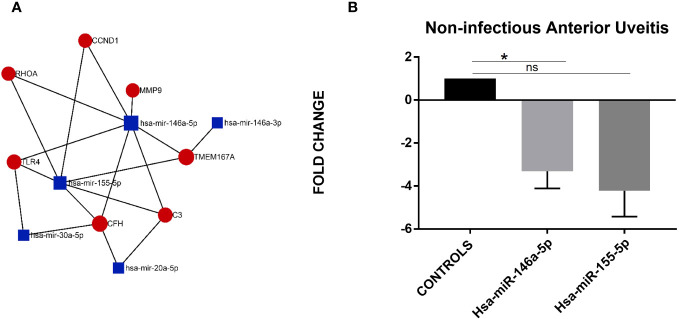
**(A)** miRNA-mRNA interaction network. This network was constructed using the differentially expressed complement component and inflammatory genes and their regulatory miRNAs. Red color indicates genes, blue color indicates miRNAs, and black lines indicate interactions between genes and miRNAs. **(B)** Differential expression based on quantitative PCR for hsa-miR-146a-5p and hsa-miR-155-5p in the non-infectious anterior uveitis category (n=10) vs. non-inflammatory controls (n=10). Data represented as mean ± SEM. *p ≤ 0.05 ns, p > 0.05.

To identify the possible regulators, we performed an *in-silico* analysis for the predicted interactions between miRNA and genes in the alternate complement component and inflammatory pathways for their possible roles in uveitis pathogenesis. The MiRNA-Gene-Network was built using the gene list and predicted interactions among them using the TargetScan database. **Figure 2** shows the miRNA–mRNA network showing their possible interactions. The center of the network represents the degree (i.e., the interaction of one miRNA with the genes around and the interaction of a gene with the miRNAs around). The key miRNAs and genes in the network had the highest degrees. Four out of five key miRNAs, namely, hsa-miR-146a-5p, hsa-miR-155-5p, hsa-miR-30a-5p, and has-miR-20a-5p, as shown in **Figure 2**, were seen to directly interact with *CFH*, *C3*, *TLR4*, and *MMP9* genes. hsa-miR-146a-5p and hsa-miR-155-5p were already reported to be differentially expressed in a study on uveitis and are known to regulate the activity of CFH for AMD and other diseases, therefore, we selected these to investigate their role in alternative complement pathway regulation in uveitis patients. quantitative PCR experiment was compared across non-infectious anterior uveitis patients and controls. Both miRNAs hsa-miR-146a-5p and hsa-miR-155-5p were downregulated in the anterior uveitis category (hsa-miR-146a-5p, AU vs control; FC-2.8 ± 0.4; ^*^ *p* = 0.03; hsa-miR-155-5p AU vs control; FC -2.1 ± 0.7 *p* = 0.2).

### Involvement of the alternative complement pathway in non-infectious posterior uveitis

**Figure 3:**
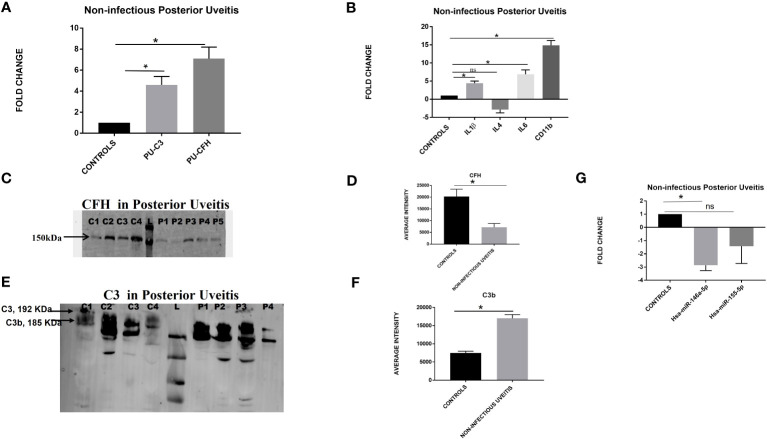
**(A)** Differential expression based on quantitative PCR for complement *C3* and *CFH* in the non-infectious posterior uveitis category (n=18) vs. non-inflammatory controls (n=18). **(B)** Differential gene expression for cytokines and *CD11b* based on quantitative PCR for the non-infectious posterior uveitis category (n=15) vs. non-inflammatory controls (n=15). **(C)** Representative Western blot of CFH in the vitreous humor of non-infectious posterior uveitis and non-inflammatory controls. **(D)** Graph showing the quantification of total CFH in non-infectious posterior uveitis and non-inflammatory controls by densitometry (NIU, n=12 and Controls, n=12). **(E)** Western blot of C3 in the vitreous humor of non-infectious posterior uveitis and non-inflammatory controls. **(F)** Graph showing the quantification of total C3b in vitreous humor of non-infectious uveitis and controls by densitometry (NIU, n=15 and Controls, n=15) **(G)** Differential expression based on quantitative PCR for hsa-miR-146a-5p and hsa-miR-155-5p in the posterior uveitis category (n=10) vs. non-inflammatory controls (n=10). For western blotting, C represents control; P, non-infectious posterior uveitis; L, protein ladder. For all the experiments, data was represented as mean ± SEM. *p ≤ 0.05, **p ≤ 0.01, ns p > 0.05.

Quantitative real-time PCR for the candidate genes, i.e., *C3* and *CFH*, in the peripheral leukocytes showed an upregulation of both *C3* and *CFH* genes (FC-4.5 ± 0.8; * *p* = 0.04 and FC-7.09 ± 1.1; *p = 0.04 respectively) in the patients with non-infectious posterior uveitis as compared to controls (**Figure 3**). Likewise, a significant upregulation of *IL1β, IL6*, and *CD11b* (*IL1β*- PU vs. control, FC-4.4 ± 0.6; *p = 0.01; *IL6-* PU vs. control, FC-6.8 ± 1.2; *p = 0.04; *CD11b-* PU vs. control, FC-14.8 ± 1.3; *p = 0.02) was observed in the posterior uveitis category, while *IL4* was downregulated (PU vs. control, FC -2.8 ± 0.9; *p* = 0.3). To further assess the complement activation at the protein level in the vitreous humor of patients with posterior uveitis, a western blot was performed for CFH and C3 in patients with non-infectious posterior uveitis and non-inflammatory controls, and a lower expression of CFH protein was observed in PU patients as compared to the controls (CFH- PU, 3199 ± 548; controls 13605 ± 1244 *p = 0.04); whereas a higher expression of C3 was observed in patients as compared to controls (C3- PU, 17027 ± 1011; controls 7510± 454 *p*= 0.04). Next, the regulation of C3 activation was assessed by performing the differential expression of hsa-miR-146a-5p and hsa-miR-155-5p. Both miRNAs, namely, hsa-miR-146a-5p and hsa-miR-155-5p, were downregulated in the posterior uveitis category (hsa-miR-146a-5p PU vs control; FC-3.3 ± 0.8 ^*^ *p- value* = 0.05, and hsa-miR-155-5p PU vs. control FC -4.2 ± 1.4 *p* = 0.4), as shown in **Figure 3**.

### Assessment of the inhibitory role of CFH on complement activation in anterior and posterior uveitis

**Figure 4:**
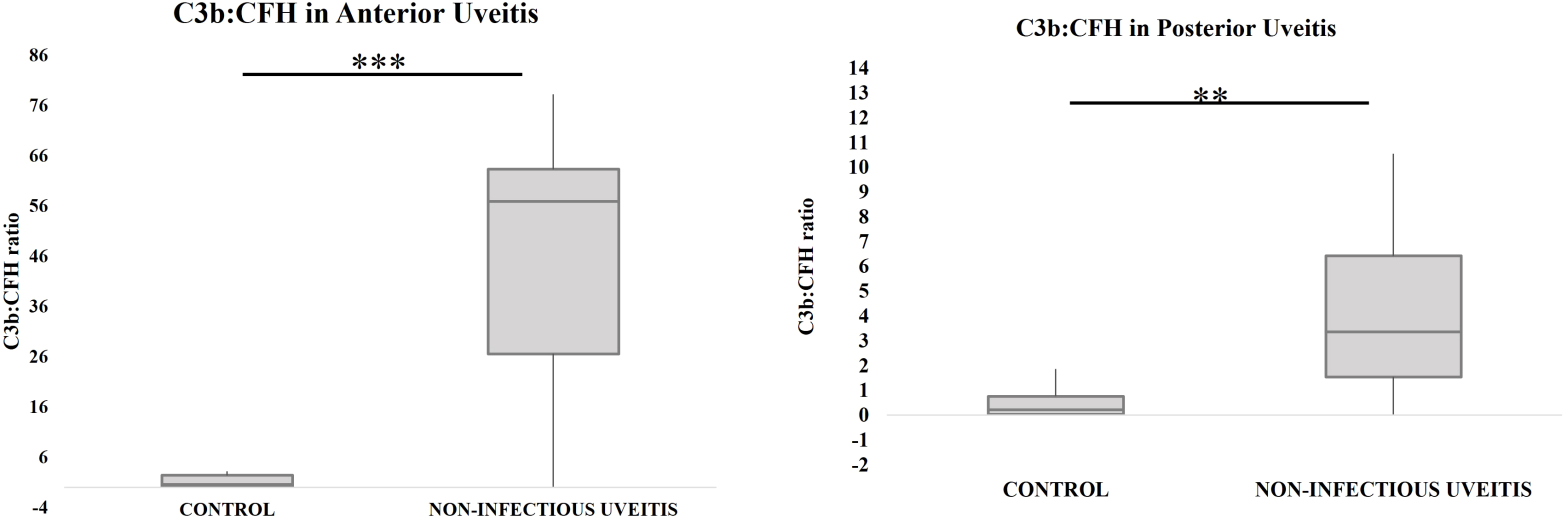
The representative box plot showing the ratio of C3b:CFH in non-infectious **(A)** anterior uveitis (n=10) and non-inflammatory controls (n=10), **(B)** posterior uveitis (n=10) and non-inflammatory controls (n=10). Data represented as mean ± SEM. **p ≤ 0.01, ***p ≤ 0.0001.

Since complement factor H is known to inhibit the excess complement activation by a feedback loop, we next computed the ratio of an activated fragment of complement C3, i.e., C3b with CFH in both non-infectious aqueous humor sample for anterior uveitis and non-infectious vitreous humor sample for posterior uveitis. As shown in **Figure 4**, the C3b:CFH ratio in patients of anterior uveitis in terms of median values (first–third quartiles) was 61.69 (31.33–71.05) as compared to the control group, 4.41 (3.93–6.22). This ratio was significantly higher for anterior NIU patients (***P =0.000004). Likewise, the C3b:CFH ratio in patients was significantly (3.05 [1.82–4.12]; **P =0.0001) higher in patients as compared to the control group, (0.55 [0.17–1.11]).

## Discussion

The human eye employs many ways to protect itself from any endogenous and exogenous insults, which not only decreases the risk of infection but also helps in preventing inappropriate immune responses, thereby plummeting the risk of inflammation–mediated retinal damage. However, ocular inflammation leading to a dysregulated immune system is still a major sight-threatening condition and concern. Uveitis represents an inflammatory condition of the uveal tract known to be associated with various etiology, such as systemic diseases, be they idiopathic or infectious. Among all forms of uveitis, anterior uveitis accounts for more than 75% of cases (20). Inflammation of the anterior uveal tract, categorized by the presence of leukocytes in the anterior chamber of the eye, is also known as iritis. When the adjacent ciliary body is also inflamed, the process is known as iridocyclitis. The immunological response during an episode of anterior uveitis might draw neutrophils to the region of inflammation, which further contributes to the formation of pus cells in the front area of the eye, and is known as hypopyon. Although neutrophils have been implicated in the pathophysiology of anterior uveitis, it is important to understand that the disease is complex and multifactorial and that the particular processes driving its development might differ across individuals (21). The presence of leukocytes in the vitreous humor and evidence of active chorioretinal inflammation are diagnostic of intermediate uveitis and posterior uveitis, respectively (1, 22). Posterior uveitis, on the other hand, can be divided into ischemic and non-ischemic posterior uveitis. Ischemic posterior uveitis represents a lack of oxygen in the afflicted region due to compromised blood flow, via vascular blockage, vasculitis (inflammation of the blood vessels), and other vascular illnesses. Ischemic posterior uveitis triggers an inflammatory response characterized by an abundance of inflammatory cells such as lymphocytes, macrophages, and neutrophils in the cellular infiltrate. Posterior uveitis that does not have an underlying ischemic cause is called non-ischemic posterior uveitis and is usually triggered by infections, autoimmune diseases, and idiopathic (unknown) factors. Lymphocytes, and in particular T cells, tend to dominate the cellular infiltration seen in these situations (23).

In the human eye, the complement pathway is seen to be constantly activated at low/basal levels and is tightly regulated by complement regulatory proteins. Previously, the complement pathway and its regulatory proteins were reported to maintain homeostasis during intraocular inflammation (13, 24). Polymorphisms in CFH, CFB, and CFI were shown to be associated with an increased susceptibility to uveitis and its many subtypes, while the C3 gene showed no association (25). However, their exact involvement in uveitis pathogenesis is yet unclear. A proteomic analysis of AH also showed differential expression of AH proteins involved in many biological functions including complement activation, humoral immune response, and proteolysis (26). Activation of the complement cascade produces mediators that directly damage cells by a consequential development of the membrane attack complex or targets the complement by activating the innate immune pathway. Furthermore, increased levels of C3, factor B, C4, and C5, as well as autoantibodies against ocular proteins, were found in AH of idiopathic uveitis. Based on this evidence, the present study hypothesized that a dysregulation of the complement system plays a role in the pathogenesis of autoimmune anterior uveitis. Since most of the samples used in the study were obtained from patients on steroid treatment. We checked if the activated complement pathway is the result of the underlying disease pathology and not due to varied treatment strategies, however, no differences with respect to the use of steroids and length of usage were noticed among our cohort. Thus, our study results might be reflective of underlying disease pathology and not a response to varied treatment strategies (12, 15, 26).

CFH is a negative regulator/inhibitor of the alternative complement pathway as it competes with FB for C3b binding and acts as a co-factor for factor I to degrade C3b to C3bi (27, 28). In contrast to our expectation, we noticed a downregulation of *C3* and *CFH* expression in peripheral leukocytes of patients with anterior uveitis as compared to controls. However, a higher expression of C3 and CFH proteins was observed in the aqueous humor, indicating that the inflammation seen in uveitis is a localized change in the anterior chamber of the eye and cannot be seen in the blood unless there is a blood-retinal barrier breakdown. Low levels of complements have also been reported to be early signs of autoimmune diseases such as systemic lupus erythematosus and rheumatoid arthritis (29–31). Thus, it is likely that the low level of complements in the serum indicates the initiation of anterior uveitis. Since complement activation leads to activation of macrophages/microglia, which further secrete anti and pro-inflammatory cytokines under stress, gene expression of the pro-inflammatory cytokines *IL1β*, *IL6*, and anti-inflammatory *IL4* was assessed along with *CD11b*, which is the marker for microglia activation. A slight upregulation of the *IL1β* and downregulation of *IL6*, *IL4*, and *CD11b* further confirmed a basal complement activation in the blood of patients with anterior uveitis.

The multiplex ELISA experiments in the aqueous humor of patients with anterior uveitis showed a definitive activation of the alternative complement pathway with significantly increased C3b in both infectious and non-infectious uveitis. A significant increase in the level of CFH in non-infectious uveitis as compared to that in infectious cases further confirmed the role of alternate complement pathway activation in NIU. A previous study by Mondino et al. also reported increased levels of activated complement fragments in the aqueous humor of eyes in patients with anterior uveitis (15). Next, we performed western blotting of aqueous humor (anterior uveitis) and vitreous humor (posterior uveitis). Upregulation of C3 and CFH in the aqueous humor was seen both by western blotting and multiplex ELISA. Since C3b complex formation is regulated by CFH, an increase in CFH could represent the underlying regulatory feedback mechanism of CFH to check further C3 activation and conversion to C3b complex.

It is also widely known that microRNAs regulate both at the level of gene transcription and protein translation, an *in-silico* analysis was performed to further understand the regulation of *C3* and *CFH* genes by miRNA. Four miRNAs, namely, hsa-miR-146a-5p, hsa-miR-155-5p, hsa-miR-30a-5p, and has-miR-20a-5p, were found directly interacting with *Complement factor H* and *C3* genes. Among these, hsa-miR-146a-5p and hsa-miR-155-5p are the known regulators of inflammatory response (32). In some studies on uveitis-associated syndromes, a reduced expression of both hsa-miR-146a and hsa-miR-155-5p was noted, however, these studies have not correlated their expression with CFH levels (18, 19). In the context of the miRNAs regulating the *CFH* gene expression, miR-146a-5p and miR-155-5p and their mimics were shown to reduce *CFH* expression in neuronal and glial cells via 3 -UTR pairing. Both miR-146a and miR-155-5p are known negative feedback-based regulators of neuroinflammation and epileptogenesis via targeting CFH (33). This explains the significant downregulation of hsa-miR-146a in anterior uveitis. Hsa-miR-155-5p was also downregulated but not significantly. Furthermore, the downregulation of their targeted genes suggests that these microRNAs do not act at the transcriptional level but at the translational level. Overall, the results from present study support that complement activation in anterior uveitis is a more localized phenomenon restricted to the anterior chamber of the eye.

In the case of posterior uveitis, upregulation of both *C3* and *CFH* genes could be an indicator of a blood-retinal breakdown (BRB). BRB is controlled by the tight junctions at the level of the retinal pigment epithelium (RPE) and becomes leaky (breaks) during inflammation. BRB causes the release of retinal autoantigens that further activate the small number of self-antigen reactive T cells that seem to have escaped thymic deletion and circulate in the periphery. This leads to an increase in number of autoreactive T cells and autoantibodies to retinal antigens (34). Based on this understanding, we speculate that it is likely that the ocular changes in the posterior uveitis will be reflected in the blood too. An upregulation of *IL1β, IL6*, and *CD11b* in the case of posterior uveitis could be due to an increased alternative complement pathway activation that further activates microglia, leading to increased secretion of pro-inflammatory cytokines such as *IL1β* and *IL6* and decreased expression of the anti-inflammatory cytokine *IL4*.

Thus, anterior and posterior uveitis seems to represent two different etiologies. A higher expression of C3b and lower expression of CFH in the vitreous humor samples of patients with posterior uveitis could be explained by the fact that a larger part of the uvea tends toward the posterior end of the eye where the inflammation is in the ciliary body, choroid, and retina. Besides in PU, both microglia and retinal pigmented epithelium are known to secrete complements and inflammatory cytokines (35), thus the reduced production of CFH by RPE cells could be due to some underlying pathological changes with defective autophagic changes. In AU, the levels of complements are majorly/entirely known to be contributed by the inflammatory cells and inflamed iris (36). A reduced expression of CFH could offer less inhibition to complement activation, leading eventually to a higher synthesis of the C3bBb complex. Previously, in the case of AMD Y402H, a single nucleotide variation in the *CFH* gene was shown to be associated with increased complement activation. The decreased CFH levels and activity also result in increased secretion of inflammatory cytokines, chemokines, and growth factors (37).

**Figure 5:**
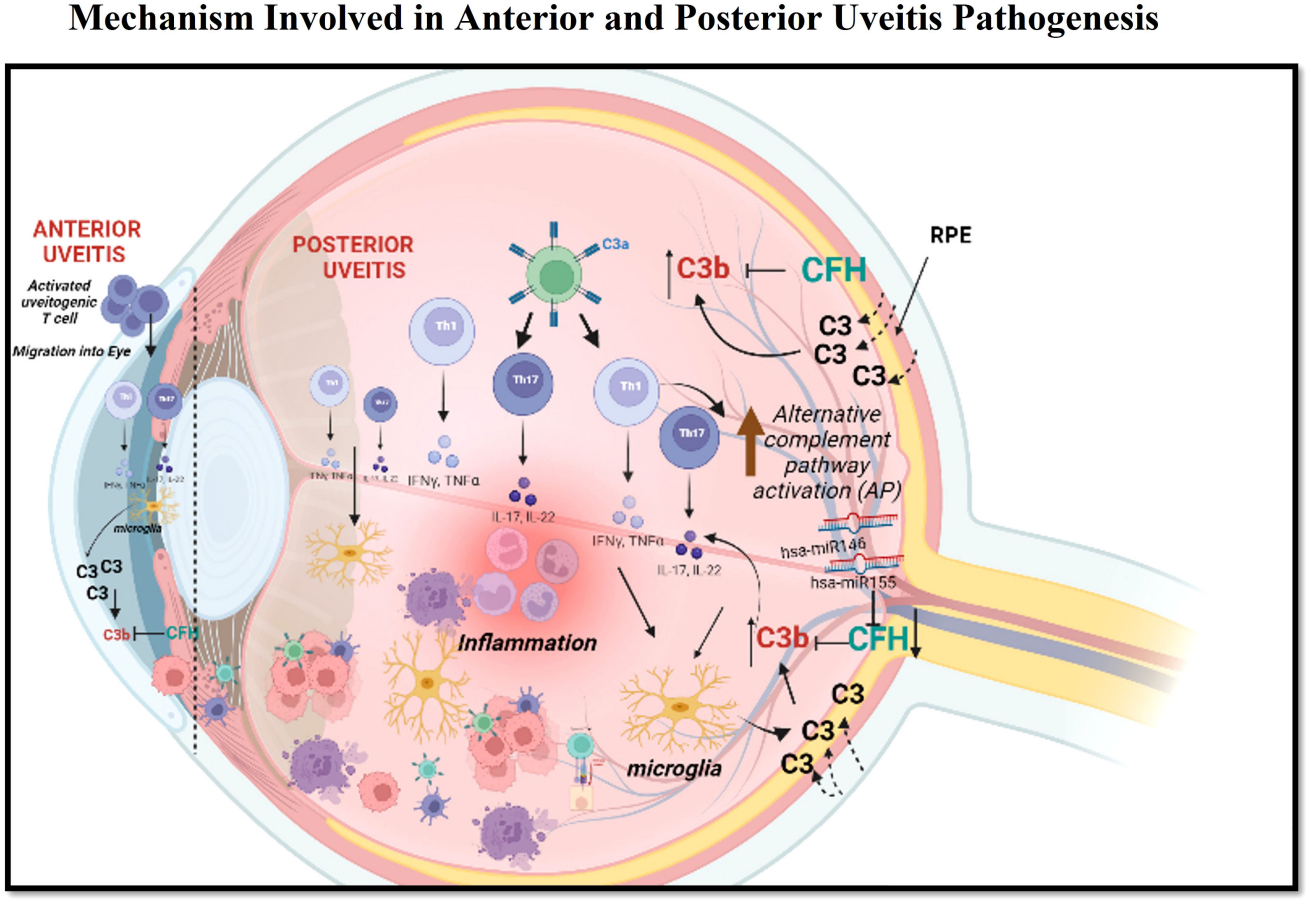
This figure demonstrates the possible mechanisms involved in the pathogenesis of anterior uveitis and posterior uveitis. In anterior uveitis, activated uveitogenic Tcells (escaped from Thymus) migrate to the eye. These uveitogenic Tcells undergo clonal expansion into Th1 and Th17 cells, which further secretes inflammatory cytokines, leading to the activation of microglia. The activated microglia further secretes C3 and other pro-inflammatory cytokines. An increase in C3 upregulates CFH as a feedback loop mechanism, which is also regulated by hsa-miR146 and hsa-miR155. In the case of posterior uveitis, the activated Th1 and Th17 cells activate microglia, which further activates the alternative complement pathway. C3b activation is regulated by CFH, which has the target site for hsa-miR146 and hsa-miR155. Apart from this, C3 is also secreted by retinal pigment epithelium (RPE). Formation of C3b and then membrane attack complex leads to an increase in the level of inflammatory cytokines and contributes to inflammation.

In the alternative complement pathway, C3b is a crucial component that helps amplify the complement response by binding to target surfaces and recruiting additional complement proteins to the site of activation (38, 39). However, uncontrolled activation of C3b can lead to tissue damage and inflammation and its lower amount can cause an immune-compromised condition. CFH binds to C3b through various domains and controls its activation. Factor H acts as a regulatory protein that helps in the degradation of C3b by promoting the cleavage of C3b into inactive fragments and preventing its accumulation in host cells. Factor H accomplishes this by binding to C3b on the surface of host cells (40, 41). Thus, an estimation of the C3b:CFH ratio in both anterior and posterior uveitis can act as a surrogate measure of immune activation. Our result showed a higher C3b:CFH ratio for both anterior and posterior uveitis patients as compared to controls. This higher range of the C3b:CFH ratio indicates the insufficient level of CFH needed to combat the aberrant activation of C3b. This further strengthens our findings on the potential role of alternative complement pathway activation in the pathogenesis of non-infectious uveitis (**Figure 5**). A further validation of complement proteins in ocular specimens from non-infectious uveitis across populations globally could help in establishing it as a predictive biomarker for disease prognosis and as a novel therapeutic target for effectively checking the inflammation and recurrence of the disease.

## Data availability statement

The raw data supporting the conclusions of this article will be made available by the authors, without undue reservation.

## Ethics statement

The studies involving humans were approved by Institutional Review Board, at L V Prasad Eye Institute, Hyderabad India. The studies were conducted in accordance with the local legislation and institutional requirements. The participants provided their written informed consent to participate in this study.

## Author contributions

IK and SS conceived the idea and served as principal investigators. IK and SS wrote the protocol. MT, SB, and PrG were co-investigators and performed clinical data collection, validation, and sub-phenotyping. PK performed most of the genomics work and protein analysis. PK and PaG performed gene and miRNA expression analysis and statistical analysis. PK, PaG, and IK analyzed the data. All authors contributed to the article and approved the submitted version.
