# Supplementary material for: Exploring the involvement of the alternative complement pathway in non-infectious uveitis pathogenesis
Source: Front Immunol. 2023 Dec 8;14:1222998. doi: 10.3389/fimmu.2023.1222998 (PMC10768558; doi:10.3389/fimmu.2023.1222998)
Supplement: Supplementary file 1 [file DataSheet_1.docx]

**Supplementary Data sheet**

**Supplementary table 1 – Demographical details of the patients included for the multiplex Elisa experiment (Aqueous humor samples)**

| **Category** | **Age** | **Gender** | **Treatment** |
| --- | --- | --- | --- |
| Controls | 58.8±2.1 | F; n =08, M; n = 02 | - |
| Non-infectious uveitis patients | 49.8±3.8 | F; n =09, M; n = 01 | Short term steroid=4, long term steroid =6 |
| Infectious uveitis patients | 46±5.1 | F; n =08, M; n = 02 | Short term steroid=3, long term steroid =7 |

**Supplementary table 2 – Demographical details of the posterior uveitis patients included for the western blotting experiment (Vitreous humor samples)**

| **Category** | **Age** | **Gender** | **Treatment** |
| --- | --- | --- | --- |
| Controls | 59±2.3 | F; n =18, M; n = 09 | - |
| Non-infectious uveitis patients | 41±3.2 | F; n =17, M; n = 10 | Active uveitis (not on steroid=19), Non active uveitis (on steroid =8) |

**Supplementary table 3 – Demographical details of the anterior uveitis patients included for the western blotting experiment (Aqueous humor samples)**

| **Category** | **Age** | **Gender** | **Treatment** |
| --- | --- | --- | --- |
| Controls | 53±3.2 | F; n =07, M; n = 03 | - |
| Non-infectious uveitis patients | 49±4.2 | F; n =07, M; n = 03 | All on long term steroid |

**Supplementary table 4- Demographical details of the patients included for the gene and miRNA expression through qPCR.**

| **Category** | **Age** | **Gender** | **Treatment** |
| --- | --- | --- | --- |
| Controls | 52.8±2.0 | F; n=19, M; n=09 | - |
| Anterior uveitis | 33.2±2.21 | F; n=16, M; n=12 | Naïve=09, long term steroid =19 |
| Posterior uveitis | 43.7±2.7 | F; n=15, M; n=13 | All on long term steroid |

**Supplementary table 5- List of primers used for gene expression.**

| **GENE** | **FORWARD PRIMER** | **REVERSE PRIMER** |
| --- | --- | --- |
| *C3* | TCACCGTCAACCACAAGCTGCTACC | TTTCATAGTAGGCTCGGATCTTCCA |
| *CFH* | TACTGGCTGGATACCTGCTC | CCTGACGGAGTCTCAAAATG |
| *IL1β* | AGCTGATGGCCCTAAACAGA | GGAGATTCGTAGCTGGATGC |
| *IL4* | CTTTGCTGCCTCCAAGAACAC | GCGAGTGTCCTTCTCATGGT |
| *IL6* | TTCGGTCCAGTTGCCTTCTC | GAGGTGAGTGGCTGTCTGTG |
| *CD11b* | CAGACAGGAAGTAGCAGCTCCT | CTGGTCATGTTGATGAAGGTGCT |
| β*-actin* | CATGTACGTTGCTATCCAGGC | CTCCTTAATGTCACGCACGAT |

| **GENE** | **FORWARD PRIMER** | **REVERSE PRIMER** |
| --- | --- | --- |
| U6 | CTCGCTTCGGCAGCACA | AACGCTTCACGAATTTGCGT |
| Hsa-miR-146a-5p | CGGCGGTGAGAACTGAATTCCA | Provided with the kit [miScript SYBR Green PCR Kit Qiagen (218173)] |
| Hsa-miR-155-5p | TTAATGCTAATCGTGATAGGGGT | Provided with the kit [miScript SYBR Green PCR Kit Qiagen (218173)] |

**Supplementary table 6 - List of primers used for miRNA expression.**

**Supplementary table 7- The details of the antibodies used and their dilutions.**

| **Antibody list** | **Conc. of protein used** | **Dilution** | **% Of gel and condition used** |
| --- | --- | --- | --- |
| Ms C3, Santacruz-sc-28294 | 15μg | 1:300 | 7.5% SDS PAGE, nonreducing condition |
|  |  |  |  |
| Ms CFH, Santacruz, sc-166613 | 30μg | 1:200 | 7.5%SDS PAGE, reducing condition |
| Anti -Ms. 680RD, Licorcatalogue no. 926-68070 |  | 1:7500 |  |
